# Dynamically actuated soft heliconical architecture via frequency of electric fields

**DOI:** 10.1038/s41467-022-30486-2

**Published:** 2022-05-17

**Authors:** Binghui Liu, Cong-Long Yuan, Hong-Long Hu, Hao Wang, Yu-Wen Zhu, Pei-Zhi Sun, Zhi-Ying Li, Zhi-Gang Zheng, Quan Li

**Affiliations:** 1 School of Physics, East China University of Science and Technology, Shanghai, 200237 China; 2 School of Materials Science and Engineering, East China University of Science and Technology, Shanghai, 200237 China; 3 School of Chemistry and Molecular Engineering, East China University of Science and Technology, Shanghai, 200237 China; 4 Advanced Materials and Liquid Crystal Institute and Chemical Physics Interdisciplinary Program, Kent State University, Kent, OH 44242 USA; 5 Institute of Advanced Materials, School of Chemistry and Chemical Engineering, and Jiangsu Province Hi-Tech Key Laboratory for Biomedical Research, Southeast University, Nanjing, 211189 China

**Keywords:** Liquid crystals, Liquid crystals, Structural properties

## Abstract

Dynamic electric field frequency actuated helical and spiral structures enable a plethora of attributes for advanced photonics and engineering in the contemporary era. Nevertheless, leveraging the frequency responsiveness of adaptive devices and systems within a broad dynamic range and maintaining restrained high-frequency induced heating remain challenging. Herein, we establish a frequency-actuated heliconical soft architecture that is quite distinct from that of common frequency-responsive soft materials. We achieve reversible modulation of the photonic bandgap in a wide spectral range by delicately coupling the frequency-dependent thermal effect, field-induced dielectric torque and elastic equilibrium. Furthermore, an information encoder prototype without the aid of complicated algorithm design is established to analogize an information encoding and decoding process with a more convenient and less costly way. A technique for taming and tailoring the distribution of the pitch length is exploited and embodied in a prototype of a spatially controlled soft photonic cavity and laser emission. This work demonstrates a distinct frequency responsiveness in a heliconical soft system, which may not merely inspire the interest in field-assisted bottom-up molecular engineering of soft matter but also facilitate the practicality of adaptive photonics.

## Abstract (web summary)

Frequency responsiveness within a broad dynamic range in adaptive systems while also reducing high-frequency induced heating remains a challenge for advanced photonics. Here, authors report a frequency-actuated heliconical soft architecture with reversible modulation of the photonic bandgap in a wide spectral range.

## Introduction

Helices and spirals are ubiquitous in nature from the molecular scale, mesoscale to macroscale and exhibit diverse physical, material, and biological properties. The dynamic, real-time and precise actuation of such structures by external stimulation, enabling the manipulation of inherent characteristics on demand, is always more desirable with both fundamental research value and contemporary significance^1–5^. Achieving helix modulation via the frequency of the applied electric field, thereby establishing a frontier topic of binary responsiveness, i.e., controlling the structure by both the strength and frequency of an electric stimulus, is the most pivotal but always a challenging topic; it is essential for high-density image recognition and storage, stereoscopic navigation, artificial neural networks, and even the embodiment of abstract mathematical concepts.

Liquid crystals (LCs), a series of versatile stimuli-responsive soft matter, are appropriate candidates that offer hope for overcoming the current challenges. LCs can generate an elegant self-organized helicoidal structure in a chiral environment^6–9^, in which molecules rotate around the helical axis and orient perpendicularly to the axis, forming the well-konwn cholesteric liquid crystal (CLC) phase. The helical LCs present selective reflection (i.e., photonic band gap, PBG) regarding impinged circularly polarized light with the same handedness sense as the helix. The central wavelength of PBG, *λ*_c_, is determined by the helical pitch length, *P*, in a certain LC system, i.e., *λ*_c_ = <*n*> *P*, wherein <*n*> is the average refractive index of LC^10–12^. Upon stimulation with an electric field, the molecules are polarized and tend to align parallel (positive LCs, Δ*ε* > 0) or perpendicular (negative LCs, Δ*ε* < 0) to the electric field, i.e., the Fréedericksz transition^13–16^.

**Fig. 1:**
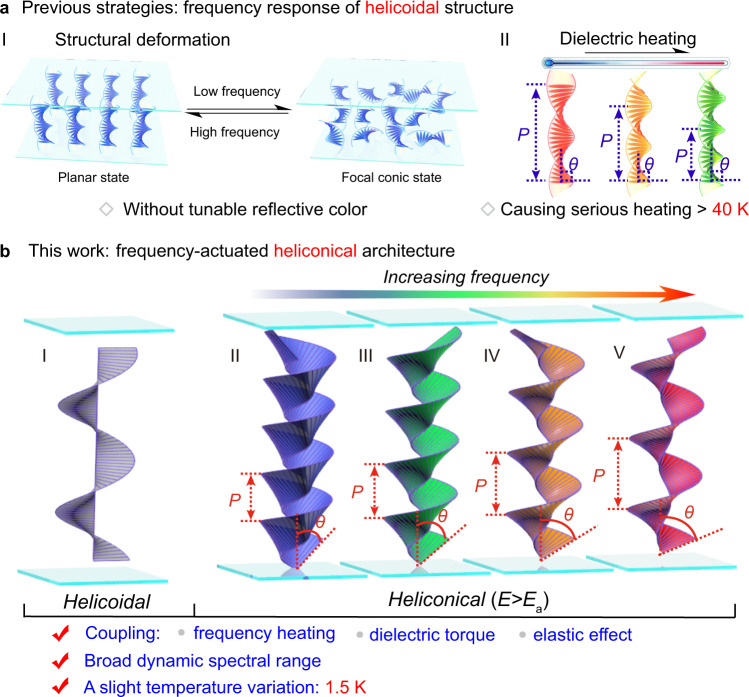
Responsiveness of chiral liquid crystals to external electric field frequency stimulation. **a** A diagrammatic sketch of the previous frequency response of (I) the structural deformation between the transparent planar state and scattering focal conic state of the helicoidal structure and (II) tuning the reflective color of the helicoidal structure through dielectric heating with an obvious thermal effect (over 40 K). The angle between the long axis of the LC molecule and the helical axis is always a right angle. **b** Schematic illustration of frequency-actuated heliconical soft architecture through delicate coupling among the frequency-dependent thermal effect, field-induced dielectric torque and elastic equilibrium at a relatively low electric field strength. (I) The helicoidal superstructure where LC molecules were perpendicular to the helical axis in the absence of an applied electric field. (II) The heliconical superstructure with an electric field signal across the LC cell of the initial frequency and strength *E* > *E*_a_, in which the LC molecules twisted around the helical axis with an oblique angle of *θ*. (II–V) The heliconical pitch length *P* and oblique angle *θ* increased with increasing frequency, resulting in wide spectral range tunability of the reflection spectrum. Herein, *E*_a_ was the threshold of the transition from the helicoidal helix to the heliconical helix.

Previous findings reveal that the arrangement of helical LCs is sensitive to both the strength and the frequency of an applied electric field, thereby indicating a possible pathway to binary responsiveness, which is commonly unattainable with any other stimuli, regardless of temperature, light, and mechanical force^7^. Generally, when the electiric field is increased, the uniformly oriented helical LCs with selective-reflection oily streaked texture are disrupted and transform to a transparent fingerprint or light-scattered focal conic texture, and even a vertical aligned unwind state, since the bend elastic constant *K*_33_ is larger than the twist elastic constant *K*_22_ in a common chiral system^17–20^. Such a transition can also be achieved by adjusting the frequency of an applied electric field in some specific series of LCs. For instance, the dynamic light scattering caused by arrangement deformation of LCs can be triggered or suppressed through low- and high-frequency LCs in an ionic LC matrix^21,22^, while the pronounced characteristic of dielectric constant interconversion depending on the frequency enables a distinct LC orientation during the Fréedericksz transition in dual-frequency LCs^23–25^. Nevertheless, the PBG effect dependent on a uniformly arranged helix is drastically weakened, therefore hampering possible photonic applications. Both the PBG and its modulation can be attained by resorting to a strong electric field with megahertz-magnitude frequency, but a troublesome trade-off is the serious heating (over 40 K) of the sample caused by the oscillation of electrons under such a high frequency^26^, thus inevitably accelerating the degradation of the devices and systems (Fig. 1a).

Recently, an electrically-induced heliconical arrangement was discovered in an LC matrix containing an optimal ratio of mesogenic dimer with a flexible alkyl as the bridged linkage^27^. Quite distinct from common helical LCs, molecules in heliconical arrangements orient obliquely at a certain angle rather than perpendicular to the helical axis due to a delicate equilibrium of bend and twist elastic energies in the system. An intriguing feature of heliconical LCs is that the PBGs can be modulated in a relatively broad dynamic spectral range covering the visible range from near ultraviolet to near-infrared (NIR) by controlling the strength of the applied electric field. Furthermore, the heliconical LCs achieve not only bias-triggered PBG but also light-stimulated interconversion between the right-handed helix and left-handed helix via adding a proper photoswitch. However, the frequency responsiveness in heliconical systems is still unattainable.

In this work, we demonstrated a frequency actuated soft heliconical architecture established in an LC matrix composed of a positive dielectric nematic LC and a specific LC dimer with two mesogenic moieties bridged by a flexible alkyl chain in a chiral environment. A PBG shift was achieved upon adjusting the frequency of the applied electric field under a relatively low electric field strength of ~0.5 V μm^−1^ over a broad spectral range (Fig. 1b) that continuously and reversibly reflected blue, green, and red wavelengths to the NIR with a slight thermal effect (the fluctuation of temperature was less than 1.5 K). This feature effectuated by a delicate equilibrium and coupling among the frequency-dependent thermal effect, field-induced dielectric torque and elastic effect the system may root in the inherent characteristic of the heliconical arranged structures, which has not been discovered so far. Such a stimulating strategy is completely distinct from the common stimuli driving the soft helical superstructure, such as light, temperature, electric field, and mechanical force^28–33^, thereby providing the possibility to achieve an always-challenging responsiveness to both the lower frequency and strength of the applied electric field, and further ensuring the compatibility of an almost-neglected thermal effect and a broad dynamic optical range. This work expands the horizon of photonic and electronic integration, networks, and systematization and provides a reliable possibility of modulating the physiological functions of biological and human organs. The frequency response also promotes further understanding of the molecular self-organized behavior, electrodynamics, and thermodynamics of soft matter under the stimulation of electric field frequency.

## Results and discussion

### Frequency actuated reflection in heliconical architecture

**Fig. 2:**
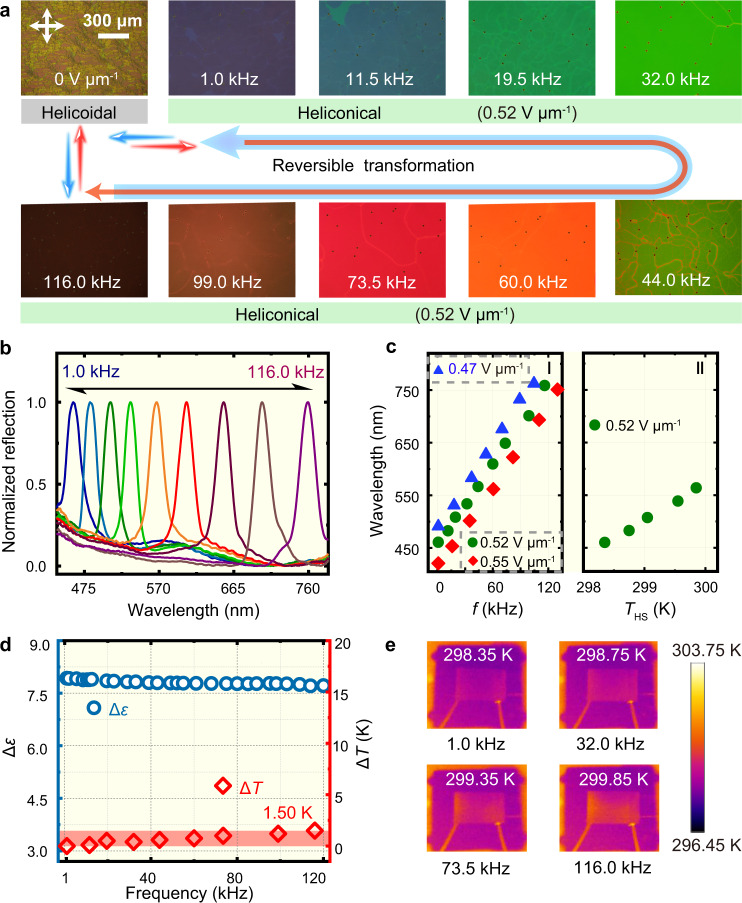
Wide dynamic spectral range of the reflection band obtained by modulating the frequency of the applied electric field. **a** Polarizing optical microscope (POM) textures of the helicoidal superstructure and the electrically induced heliconical superstructure accompanied by different reflection colors when a fixed electric field strength (i.e., 0.52 V μm^−1^) with increasing frequency from 1.0 to 116.0 kHz. Orthogonal double-arrows means the crossed polarizers. The scale bar is 300 μm. **b** The corresponding reflection spectra of the POM textures when the electric field strength (i.e., 0.52 V μm^−1^) was constant. **c** Frequency-dependent central wavelength of the reflection bands at various electric field strengths (I) and the evolution under a direct change in temperature (*T*_HS_) controlled with a hot stage from 298.35 to 299.85 K under fixed electric field strength and frequency (i.e., 0.52 V μm^−1^ and 1.0 kHz) (II). **d** Almost unchanged dielectric anisotropy Δ*ε* and the slight change in sample temperature (Δ*T*) of 1.50 K at 0.52 V μm^−1^ under increasing frequency from 1.0 to 116.0 kHz. Δ*T* was defined as *T*–*T*_0_, and the initial temperature *T*_0_ of the sample at 1.0 kHz was 298.35 K. **e** Infrared temperature measurement of the sample with increasing electric field frequency by a thermal imager. The sample was located in the center of the rectangular area. Source data are provided as a Source Data file.

To achieve a broader dynamic spectral range of the reflection band by modulating the frequency of an applied electric field at room temperature, the material system consisted of a mixture of nematic LC E7 with a lower clearing point (333.15 K), LC dimer CB7CB with two cyano-biphenyl-based mesogens bridged by n-heptyl, and a suitable amount of chiral agent R811 (Supplementary Fig. 1). CB7CB showed a uniaxial nematic phase between 390.45 K and 377.25 K, sandwiched between the isotropic and the twist-bend nematic phase. Prior work demonstrated that LC molecules arranged into a common periodic helicoidal superstructure at initiation, where the molecules were locally oriented perpendicular to the helical axis. The helicoidal structure would transform to an asymmetric topological heliconical structure by applying an electric field with appropriate strength between the threshold (*E*_a_) and the unwinding (*E*_b_) fields (Supplementary Fig. 2), owing to the coupling and competition between the elastic effects and the dielectric torque^34,35^ in the chiral LC system. When the electric field was absent, the focal conic optical texture of the sample was observed initially, manifesting the common helicoidal arrangement of LCs, followed by a transition to a uniform bluish reflection texture with a central wavelength of 460 nm after suffering a square wave electric field with a frequency of 1.0 kHz and an amplitude of 0.52 V μm^−1^, confirming the formation of a heliconical arrangement. Further observations exhibited the evolution of the texture with the modulation of the electric field frequency; these observations were quite distinct from those in any relevant previous reports^26,36^. Significantly, continuously raising the frequency while maintaining the strength of the electric field caused a broad redshift in the reflection band of more than 300 nm, spanning almost the entire visible band, to the central wavelength of 760 nm as the frequency was 116.0 kHz. The corresponding reflection color successively transformed into cyan (11.5 kHz), green (32.0 kHz), red (73.5 kHz) and almost NIR (116.0 kHz) (Fig. 2a, b and Supplementary Movie 1), thereby enabling the three primary color-based image displays to be manipulated by the frequency of the electric field. Additionally, similar spectral dynamic behaviors were further confirmed by modulating the frequency of the applied electric field with either a higher or lower electric field strength (Fig. 2c). It is remarkable that the frequency responsive reflection spectrum of such heliconical structure in Fig. 2 was measured from 460 to 760 nm mainly covering the visible reflection band for conveniently intuitive observation and practical application. However, such reflection band could further shift to nearly 1000 nm by increasing the frequency to about 222.0 kHz (Supplementary Fig. 3).

Such specific frequency dependent reflection color modulation subconsciously inspired us to consider two major possible factors, i.e., the possible inherent dielectric frequency dependent characteristics of the LCs and the dielectric thermal effect caused by the high frequency of the electric field. Nevertheless, the dielectric constants of the LC mixture, pure CB7CB or E7 were almost constants over the frequency range of electric field applied in the experiments (1.0–120.0 kHz), and exhibited the positive characteristics (Fig. 2d and Supplementary Fig. 4), indicating that the dramatic shift of reflection cannot be attributed to the frequency-induced change of the dielectric constant. Additionally, an imperceptible increase in the sample temperature of only ~1.50 K was detected by a thermal imager (Fig. 2d, e) after applying a 116.0 kHz electric field across the LC cell. Although the reflection band of the heliconcial superstructure was sensitive to the temperature^37^, this raised temperature was not actually the root cause of the broad dynamic spectral range. First, the optical texture was almost unchanged as the LC was directly heated by 1.50 K through an accurate hot stage in either the absence or presence of an electric field lower than the threshold (Supplementary Fig. 5). When the electric field exceeds the threshold (*E*_a_) and fixed at 0.52 V μm^−1^ and 1.0 kHz, directly heating the sample at an elevated temperature of 1.50 K resulted in a very limited red-shift (about 100 nm) in the corrsponding reflection spectra (Fig. 2c). Thus, the change of tempertature induced by high frequency of applied electric-field was supposed not the only reason for such a wide spectral shifting from 460 to 760 nm.

In fact, the phenomenon revealed a delicate coupling among the frequency thermal effect, the dielectric torque, and the equilibrium of the bending and twisting elastic interaction, that leaded to the frequency-responsive wide range reflection spectrum. To elucidate such coupling, the frequency- and temperature-dependent elastic effect and the corresponding reflection spectrum were deduced upon the combination of experimental results and the classical elastic energy model. Prior theoretical work^27^ indicated a significant dependence of the evolution of the reflection band on the pitch length *P* and oblique angle *θ* of the heliconical structure (shown in Fig. 1b), which were further determined by the bend elastic constant *K*_33_ and the ratio of the bend and twist elastic constants *σ* = *K*_33_/*K*_22_ based on Supplementary Eqs. (6)–(8).

According to the Frank elastic theory (see Supplementary Eqs. (1)–(8)) and Berreman’s 4 × 4 matrix method^38–40^ (Supplementary Eqs. (9)–(20)), which have been widely used for analyzing optical properties of LC, the reflection spectra at certain applied electric fields can be simulated simultaneously (Supplementary Fig. 6a, b). Furthermore, the *K*_33_ and *σ* of the heliconical system in the corresponding situations were obtained by fitting simulated data to experimental data. Supplementary Fig. 6c, d shoeds similar monotonic and exponential growth of *K*_33_ and *σ* at a constant electric field strength (0.52 V μm^−1^) and an increased frequency. It is noteworthy that after applying an invariable electric field with the settled strength and frequency, the fitted *K*_33_ of the heliconical system displayed a significantly lower variation if only the temperature is increased, despite having the same monotonic growth tendency (Supplementary Fig. 7). The finite-difference time-domain method^41^ was also applied to simulate the reflection spectra of the heliconical system and showed a satisfactory agreement with the results obtained by Berreman 4 × 4 matrix method, indicating the feasibility of the Berreman 4 × 4 matrix method for optical analysis in the heliconical structure (Supplementary Fig. 8). To further verify the results, the dependency of *K*_33_ on the frequency regarding to pure CB7CB and E7 were also determined according to Supplementary Eqs. (21)–(23), respectively. The bend elastic constants of CB7CB and E7 were enhanced with increasing frequencies (Supplementary Fig. 9). CB7CB played a dominant role to the reduction of *K*_33_ in the mixture, ensuring the formation and stability of such a heliconical system during the modulation of frequency, and therefore presenting a frequency controllable band shift of the reflection within a broad spectral range. *K*_33_ of CB7CB exhibited a conspicuous rising with the increase of the frequency as the temperature was closer to the phase transition between the twist-bend nematic and nematic phase (Supplementary Fig. 10). Consequently, the frequency dependence of the PBG within such a broad dynamic range was probably attributed to a delicate equilibrium and coupling among the frequency-dependent thermal effect, field-induced dielectric torque and elastic effect of the system, in which the elastic effect was not only affected indirectly by high-frequency heating, but also presented a direct dependency on the frequency.

Moreover, *K*_33_ and *σ* related to the electric field frequency were fitted with exponential Supplementary Eqs. (24) and (25), wherein parameter *f*_s_ denoted the transition frequency of the system from the heliconical to the helicoidal structure. Theoretically, the helical system would transform from the heliconical to the helicoidal structure as the frequency exceeded *f*_s_, which was corroborated experimentally at 342.0 kHz when the electric field was 0.52 V μm^−1^ (Supplementary Fig. 11a). The corresponding relative pitch length, defined as the ratio of the current and the original pitch length *P*/*P*_0_, gradually increased to ~0.32, and the oblique angle *θ* increased to ~30°. As the frequency exceeded *f*_s_, the oblique angle abruptly jumped to 90°, while the pitch length transited to the original value (Supplementary Fig. 11b). Furthermore, the frequency-related helical pitch (*P*), oblique angle (*θ*), unwound electric field (*E*_b_) and threshold electric field (*E*_a_) required for generating heliconical arrangements under different electric field strengths were deduced (Supplementary Fig. 12). Based on the helical pitch and oblique angle expressed by Supplementary Eqs. (26)–(29), respectively, the relationship between the central wavelength of the reflection band and the frequency of the applied electric field can be described by Supplementary Eqs. (30)–(36), which was further confirmed by the evolution of the central reflection wavelength with increasing electric field frequency when the strength of the electric field was different (Supplementary Fig. 13). Notably, the central wavelength was proportional to the strength of the electric field when the frequency was invariable, which corroborated the aforementioned intrinsic coupling of the frequency responsiveness of the heliconical system from another perspective.

The similar frequency dependency of the reflection spectrum was observed as the nematic LC used herein was replaced by another nematic LC 5CB with the positive dielectric constant (Supplementary Fig. 14). Therefore, we predicted that such frequency-dependency may be an intrinsic characteristic of the heliconical arranged system, rather caused by the common dielectric anisotropy of the system.

### Information encryption and encoding

**Fig. 3:**
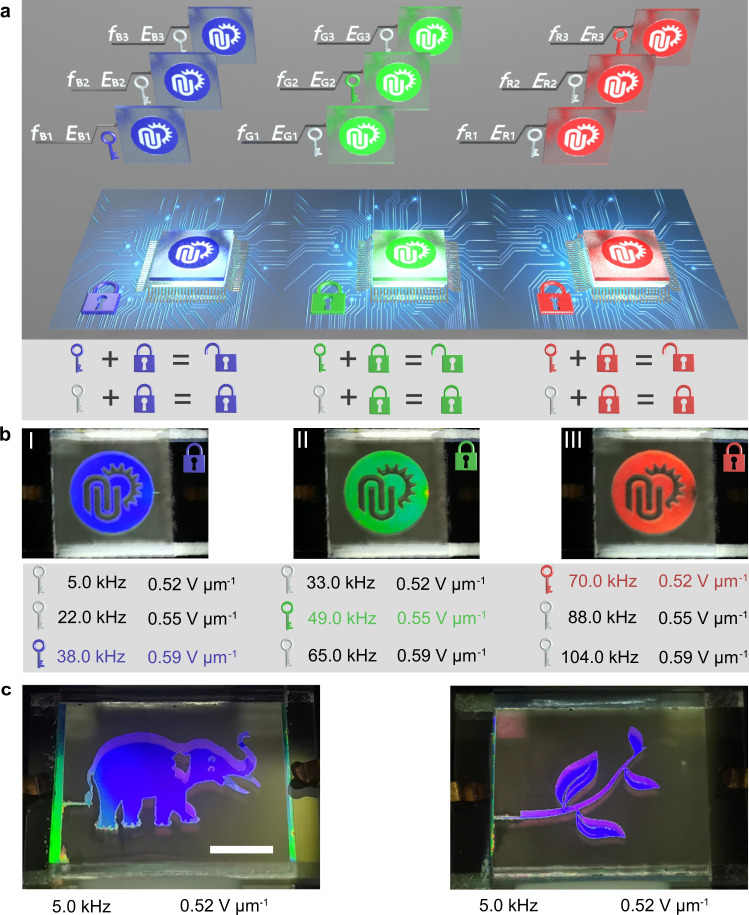
Active information encryption with the required images. **a** Schematic diagram of active information encryption by setting one of a series of electric fields with different strengths and frequencies corresponding to a certain reflection color image as the encryption key of an information. **b** Images with a certain reflection color were obtained through a series of electric fields. **c** The images with a three-dimensional visual effect are shown with a slightly oblique viewing angle. The scale bar represents 5 mm.

Any desired images could be presented and hidden by applying and removing an electric field across the cell with the patterned electrodes corresponding to the images. A predefined logo with a sharp edge, displaying continuous dynamic modulation of the reflection color, covering red, green and blue, was achieved upon solely modulating the strength or the frequency of the electric field. And moreover, the coupled modulation of the strength and frequency also enabled the unique responsiveness with the applied electric-field, which promoted a feasible active information encryption without a complicated algorithm design. As illustrated in Fig. 3a,the logo with similar reflection color could be achieved in the cases of a series combinations of strengths and frequencies, for instance, the blue color could be generated by a field of 0.52 V μm^−1^ with the frequency of 5.0 kHz, or a field of 0.59 V μm^−1^ with the frequency of 38.0 kHz. This meant that a defined image with a similar reflection color could be generated with many kinds of strength-and-frequency combinations of applied electric field, therefore exponentially enhancing the confidentiality (Fig. 3b and Supplementary Movie 2). In detail, an encryption could be achieved conveniently by predefining a strength-and-frequency combination as a correct key, and therefore the counterpart decryption was accomplished by entering the preset key. A further enhancement on the encryption can be considered by adopting the reflection color information of the emersed image. And moreover, a three-dimensional visual effect of the image was found in the case of a slight oblique viewing angle, which was probably caused by the heliconical structure and the displacement of the image reflected from the LC matrix and the cell substrate, respectively (Fig. 3c). This attribute reinforced the encryption from the changing of viewing angle.

**Fig. 4:**
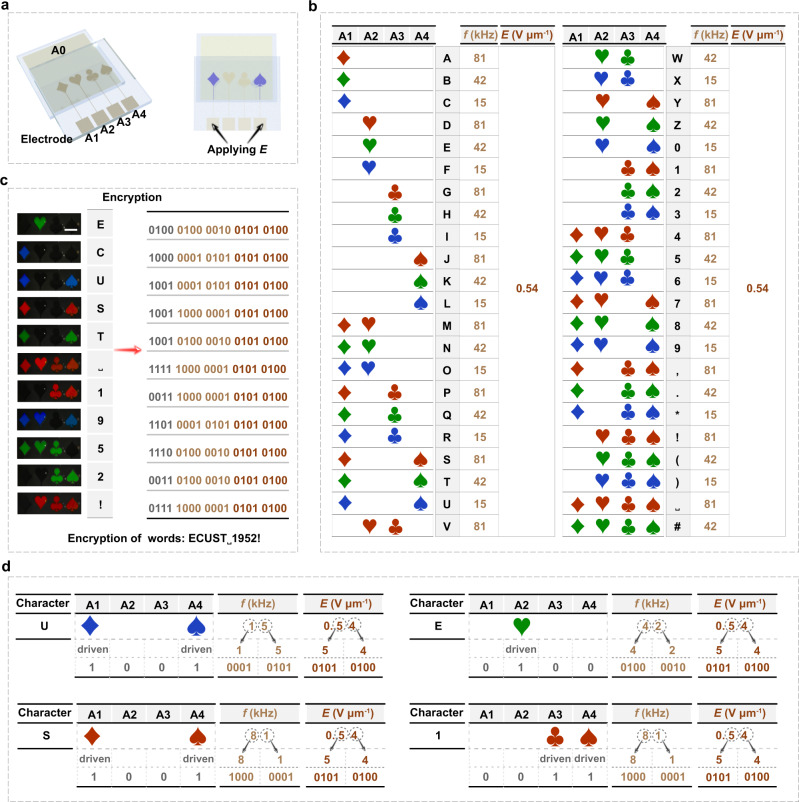
Encoding and decoding process of the information encoder. **a** Schematic diagram of pattern design for information encoding. Four active areas with different Poker patterns were predesigned on the upper substrate of a single LC cell by etching the corresponding patterned indium tin oxide (ITO) on one substrate (A1, A2, A3 and A4) while a planar ITO (A0) on the other substrate as a common electrode. These active areas can be independently driven by an applied electric signal (*E*). **b** The encoding table describing the one-to-one correspondence between the specific patterns and the characters. **c** The experimental patterns representing a series of words “ECUST˽1952!” were encrypted with binary codes. The scale bar is 5 mm. **d** The decryption rules of the characters (such as “U”, “S”, “E” and “1”) were formulated with 20 bits binary codes, where the first 4 bits represented four active areas from the left to right, the driven area denoted “1” and the non-driven area was “0”. The frequency of driven signal was expressed by the middle 8 bits, in which the former and latter 4 bits represented the ten digits and single digits of the frequency respectively. In addition, the final 8 bits denoted the field strength of driven signal, in which the deciles and percentiles were the former and latter 4 bits. “˽” means the “space” character.

To further explore the potential application of such frequency actuated soft heliconical system, an information encoder prototype was rationally designed to analogize an information encoding and decoding process. As schematically shown in Fig. 4a, four active areas with different Poker patterns were predesigned in a single LC cell with the corresponding patterned indium tin oxide (ITO) on one substrate (A1, A2, A3 and A4) while a planar ITO on the other substrate (A0) as a common electrode. These active areas can be independently modulated by an applied electric signal, which enabled the information encoding with the combination of the sequence and color of different Poker patterns. For instance a combination of bluish diamond and spade pattern denoted the capital character “U”, which was encoded by applying a 0.54 V μm^−1^ and 15 kHz signal independently on A1 and A4. If signal frequency was increased to 81 kHz while the field strength was invariable, the diamond and spade changed to reddish, which represented to the character “S”. Similarly, a greenish heart displayed by applying a 41 kHz signal with maintained field strength on A2, achieving the encoding of character “E”. With the various combinations of four Poker patterns and their reflection colors, an encoding table describing the one-to-one correspondence between the specific patterns and the characters were designed and demonstrated in Fig. 4b, which enabled the encryption of a series of words ‘ECUST˽1952!’ as shown in Fig. 4c and Supplementary Movie 3. To decrypt the corresponding information, a set of decryption rules were formulated with 20 bits binary codes considering on both the convenience of optical fiber transmission and the confidentiality (Fig. 4d). Specifically, as illustrated in Fig. 4d, the first 4 bits represented four active areas from the left to right, the driven area denoted “1” while the non-driven area was “0”. The frequency of driven signal was expressed by the middle 8 bits, in which the former and latter 4 bits represented the ten digits and single digits of the frequency respectively, and in addition, the final 8 bits denoted the field strength of driven signal, in which the deciles and percentiles were expressed respectively by the former and latter 4 bits. Each series of 20 bits binary codes implied a decryption manner to display the correct patterns and colors, and thereby decoding the encrypted information according to the encoding table shown in Fig. 4b. Importantly, the frequency modulated heliconical system with an invariable field strength ingeniously avoided arrangement deformation of LCs induced by the fringing field effect resulting from the bias differences between two active areas. Such encryption and decryption processes were independent on both the complicated device design and tedious algorithm compared to the common strategy used so far, and moreover the designed encoding table and the decryption rules encrypted the information from both the transmission, encryption and decryption processes, therefore significantly enhancing the confidentiality with a more convenient, easier, and cost-less way. The encryption can be further enhanced by introducing the reflection color differences among the Poker patterns through properly frequency actuation.

### Spatially controlled soft photonic microcavity

**Fig. 5:**
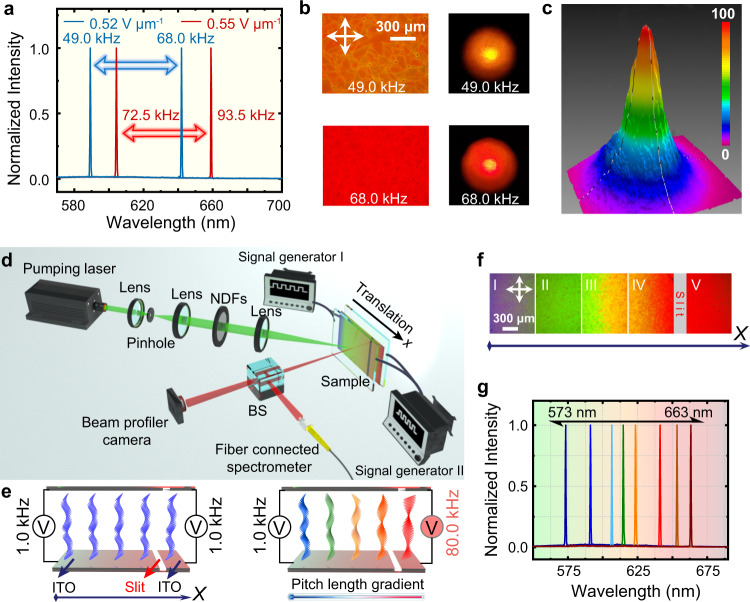
Dynamic tunable laser emission in heliconical LC through the frequency of the electric field. **a** Laser emission spectra from the heliconical superstructure. **b** Corresponding POM textures (left) and laser emission spots (right). **c** Gaussian distribution of the laser emission intensity profile. **d** Schematic diagram of the experimental setup for characterizing laser performance (see “Methods”). Neural density filters (NDFs), Beam splitter (BS). **e** Two independent electric field signals were applied to the specially designed sample with signal generators I and II. The upper and lower electrode layers (i.e., indium tin oxide, ITO) on the glass substrates of the sample were separated into two discrete regions with nonconductive slits. When the applied electric field strengths and frequencies of the two regions were both the same (i.e., 0.50 V μm^−1^, 1.0 kHz), the sample presented a uniform bluish reflection color. When the frequency of the electric field was adjusted to 80.0 kHz on the right side while the frequency of the left side remained unchanged, the entire sample generated a temperature gradient distribution with a gradual, rainbow-like change in reflection colors from left to right. **f** The corresponding POM textures. **g** Laser emission spectra covering 573 to 663 nm, which could be modulated by translating the sample on the *x*-axis to generate the laser of the cell. Here, the cell gap was kept at 96 µm thick for obtaining the preferable laser properties. Source data are provided as a Source Data file.

The helical arrangement of LCs furnishes the conspicuous photonic bandgap resembling the photonic crystal, thus enabling specific laser emission when pumped by external light excitation after loading a small amount of gain medium in LCs. Lasers commonly at the long-wavelength band edge (LWBE) provided that the fluorescence spectrum of the gain medium overlaps with the bandgap of the helical LCs, thereby achieving a specific continuous tunability of the emission wavelength through various stimuli, e.g., temperature, light irradiation, and applied bias^42^. To corroborate the molecular arrangement of frequency-responsive heliconical LC, laser emission and the relevant dynamic modulation through the frequency of the electric field were explored. A yellowish reflection texture was observed as a 0.52 V μm^−1^ and 49.0 kHz electric field was applied across the sample, thereby generating yellow laser emission with a sharp spectrum at the corresponding LWBE, i.e., 590 nm (Supplementary Fig. 15a), which manifested a satisfying photonic bandgap of the heliconical structure. As expected, the color changed to red when the frequency increased to 68.0 kHz while maintaining the strength of the electric field, thus inducing the shift in the laser emission to ~642 nm and presenting a bright reddish color (Fig. 5a, b). The emission profile of laser spots was in accordance with a typical Gaussian distribution (Fig. 5c), reconfirming the structural transformation triggered by frequency modulation. Similarly, the laser emission at 604 nm was tuned to 659 nm by increasing the frequency from 72.5 to 93.5 kHz while maintaining the electric field at 0.55 V μm^−1^. Continuously raising the frequency to 105.0 kHz could inhibit the laser emission because the reflection band shifted beyond the fluorescence band of the laser dye (Supplementary Fig. 15b); that is, the optical gain in this case was too weak to generate emission. The laser wavelength could be recovered by tuning the frequency to the original value or even increasing the frequency and simultaneously enhancing the strength of the electric field. For instance, laser emission at ~590 nm could be modulated by enhancing the electric field from 0.52 to 0.55 V μm^−1^ and simultaneously increasing the frequency from 49.0 to 67.0 kHz, which provided an appropriate prototype for binary responsive photonic chips or prospective binary control systems. The pumping threshold of the laser emission is 1.55 µJ per pulse, determined by the change of emission spectra during the increasing of pumping energy (Supplementary Fig. 16).

Moreover, a spatial gradient distribution of pitch length in the heliconical system was achieved by applying two independent electric fields with the same strength but different frequencies on two prescribed discrete ITO regions on the single cell (Fig. 5d, e). The ITO electrode on each substrate was divided into two parts by etching a specially designed slit with the same width. Distinct from the common pitch gradient generated by a continuous gradient of either the temperature or the surficial anchoring using a wedge LC cell with a certain prescribed wedge angle^43,44^. Herein, the frequency heating effect and the combination with elastic equilibrium enabled a special spatial pitch gradient of heliconical LCs. The rainbow-like reflection colors (Fig. 5f) were acquired by applying a relatively low frequency electric field (i.e., 0.50 V μm^−1^, 1.0 kHz) on left side and a higher frequency (i.e., 0.50 V μm^−1^, 80.0 kHz) on the right side, forming a temperature gradient due to the difference in electric field frequency between the two sides, therefore resulting in the gradient distribution of pitch length along the direction of the frequency difference, i.e., the *x*-axis. By transversely moving the sample, the laser emission can be reversibly modulated from 573 to 663 nm (Fig. 5g), and furthermore, the band width of the laser was maintained at ~0.5 nm during the entire modulation process. This rainbow-like reflection disappeared and transformed to a uniform bluish color when the higher frequency (i.e., 80.0 kHz) was switched to a lower frequency (i.e., 1.0 kHz). The pitch length and any desired pitch distributions can be readily configured and removed upon an advisable setting of electric field frequency applied on both sides or by the specific design of the shape of the etched slit, thus exploiting a strategy toward smart manipulation of soft helical system, not merely limited on the common pitch elongation and shortening, but the preferable distribution with prescribed gradient of the pitch length.

In conclusion, a frequency-actuated soft photonic superstructure with a heliconical arrangement was achieved based on a specially designed nondual-frequency cholesteric system composed of a common nematic LC and an LC dimer. Such a soft superstructure featured a reversible modulation of PBG within a relatively broad spectral range, covering the blue, green, red, and near infrared reflection bands, via adjustment of the frequency of the applied electric field. The frequency dependence of the PBG within such a broad dynamic range was probably attributed to the delicate coupling among the frequency-induced heating, dielectric torque, and elastic equilibrium of the bend and twist deformation of the heliconical system, which was quite distinct from the previously reported modulations using any electric field, temperature, light, or mechanical force. The electric responsiveness of the heliconical LCs to both the electric field strength and the frequency (i.e., binary responsiveness) has enabled presentative functions toward a series of applications on information encryption and decryption. Furthermore, a predefinition of the helical pitch length of LCs with a prescribed gradient distribution was achieved in soft matter based on the frequency differences between two applied electric fields, thereby promoting the occurrence of excited prototypes of photonic applications, such as the tunable laser emission demonstrated herein. This work provides a specific strategy to efficiently control, program and set soft materials and relevant systems, further developing areas like photonics, soft matter, physics, and even biological soft systems and medical sciences.

## Methods

### Material preparation

The chiral LC mixture consisted of 53.1 wt% commercial nematic LC (E7, Slichem Co., Ltd, China), 43.4 wt% LC dimer (CB7CB, synthesized in the lab; the concentration ratio of E7 to CB7CB was ~55:45) and 3.5 wt% chiral agent R811 (Merck, Germany) to appropriately enhance the twist elastic effect of the system. The LC director preferentially arranged in a parallel orientation to the electric field because the E7 and LC dimer CB7CB possess positive dielectric anisotropies. The mixture was stirred sufficiently at 373.15 K (above the clearing point 348.15 K) and injected into the LC cell with a gap of 12 µm. The cell was prepared by two ITO glass substrates with parallel alignment. Then, the sample was cooled to 298.15 K at a precisely-controlled rate of 0.3 K min^−1^ on a hot stage (LTS120E, Linkam, UK). Furthermore, 0.5 wt% of the laser dye 4-dicyanomethylene-2-methyl-6-(4-dimethylaminostyryl)-4H-pyran (DCM, Sigma-Aldrich, USA) was dispersed into the CLC mixture for laser emission. The cell gap was kept at 96 µm thick to obtain the preferable laser properties. The aforementioned process was repeated to prepare the sample for lasing.

### Characterization

The sample was specially designed to ensure that two independent electric field signals could be applied, and the electrode layers of the sample on the glass substrates were separated into two discrete regions with a nonconductive band by sculpting the nonconductive sections (Fig. 5e). Two independent square wave signals were applied to the sample, whose electric field strengths remained the same and the frequencies were adjustable; they were generated via two signal generators (AFG3022, Tektronix, USA) that passed the signal through the corresponding signal amplifiers (A600, FLC Electronics, USA). The POM textures were captured by a digital camera (DS-U3, Nikon, Japan) coupled with polarized optical microscopy (LVPOL 100, Nikon, Japan), while the reflection spectra were recorded by a fiber connected to a spectrometer (ULS2048, Avantes, the Netherlands, resolution: ~0.3 nm). The infrared photographs were collected by a thermal imager (TIS65, FLUKE, USA).

### Lasing emission

The laser emission was induced by a 532 nm second-harmonic Q-switched Nd:YAG (neodymium-doped yttrium aluminum garnet) pulsed laser (Dawa 200, Beamtech, Canada) with an 8-ns pulse width at 10 Hz (Fig. 5d). To obtain the uniform distribution of pump energy, the pump beam was filtered and expanded through a small hole sandwiched between a pair of lenses to obtain the uniform distribution of pump energy with the intensity tuned by a neural density filter. A convex lens was set to focus the beam on the sample at an oblique angle of incidence (~45°) for detecting the laser emission. The sample was located in a precision linear displacement platform to facilitate the detection of laser emission from different positions. The emission wavelength was measured by the fiber connected to a spectrometer, and the intensity distribution was analyzed by a beam profiler camera (SP620U, Spiricon, USA).

### Statistics and reproducibility

No statistical method was used to predetermine sample size. No data were excluded from the analyses.

### Reporting summary

Further information on research design is available in the Nature Research Reporting Summary linked to this article.

## Supplementary information

Supplementary Information

Description of Additional Supplementary Files

Supplementary Movie 1

Supplementary Movie 2

Supplementary Movie 3

Lasing Reporting Summary

## Data Availability

The data generated in this study are provided in the paper and the Supplementary Information. Source data are provided with this paper.

## Source data

Source Data
