# Supplementary material for: Dynamically actuated soft heliconical architecture via frequency of electric fields
Source: Nat Commun. 2022 May 17;13:2712. doi: 10.1038/s41467-022-30486-2 (PMC9114134; doi:10.1038/s41467-022-30486-2)
Supplement: Supplementary file 1 — Supplementary Information [file 41467_2022_30486_MOESM1_ESM.pdf]

Supplementary information

Dynamically Actuated Soft Heliconical Architecture via Frequency of Electric Fields

Binghui Liu[1,2,6], Cong-Long Yuan[1,2,6], Hong-Long Hu[3,6], Hao Wang[4], Yu-Wen Zhu[2], Pei-Zhi Sun[2], Zhi-Ying Li[2], Zhi-Gang Zheng[1,2,*] & Quan Li[4,5,*]

[1] School of Physics, East China University of Science and Technology, Shanghai 200237, China. *E-mail: zgzheng@ecust.edu.cn (Z.-G.Z.)

[2]School of Materials Science and Engineering, East China University of Science and Technology, Shanghai 200237, China

[3]School of Chemistry and Molecular Engineering, East China University of Science and Technology, Shanghai 200237, China

[4]Advanced Materials and Liquid Crystal Institute and Chemical Physics Interdisciplinary Program, Kent State University, Kent, OH 44242, USA

[5]Institute of Advanced Materials, School of Chemistry and Chemical Engineering, and Jiangsu Province Hi-Tech Key Laboratory for Biomedical Research, Southeast University, Nanjing 211189, China. *E-mail: quanli3273@gmail.com (Q.L.)

[6]These authors contributed equally: Binghui Liu, Cong-Long Yuan, Hong-Long Hu

I. Supplementary Figures

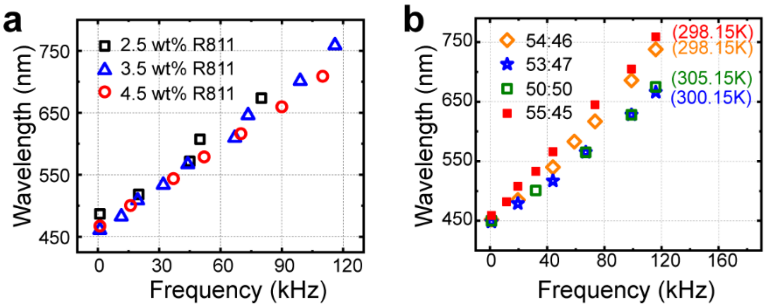

Supplementary Fig. 1 Optimizing the concentrations of the composites in the CLC mixture. **a** The ratio of E7 and CB7CB in these mixtures were remained at 55: 45 and the temperature were 298.15 K, while the concentrations of R811 were 2.5 wt%, 3.5 wt% and 4.5 wt% corresponding to the driving electric fields 0.40 V μm^{-1}, 0.52 V μm^{-1} and 0.59 V μm^{-1}, respectively, in order to ensure the initial central wavelength of reflection band located between 450 nm and 500 nm. **b** The concentration ratio of E7 and CB7CB was optimized at 0.52 V μm^{-1} when the concentration of R811 was 3.5 wt%. The widest modulated range of reflection band existed at the room temperature (around 298.15 K) when the concentration ratio was 55: 45. Specially, the spectral ranges corresponding to the ratios 53: 47 and 50: 50 were obtained at 300.15 K and 305.15 K, respectively, which were the optimal temperatures to the reflectance spectra of these ratios, in other words, the modulated ranges of reflection band would narrow or even disappear when the temperatures dropped to 298.15 K.

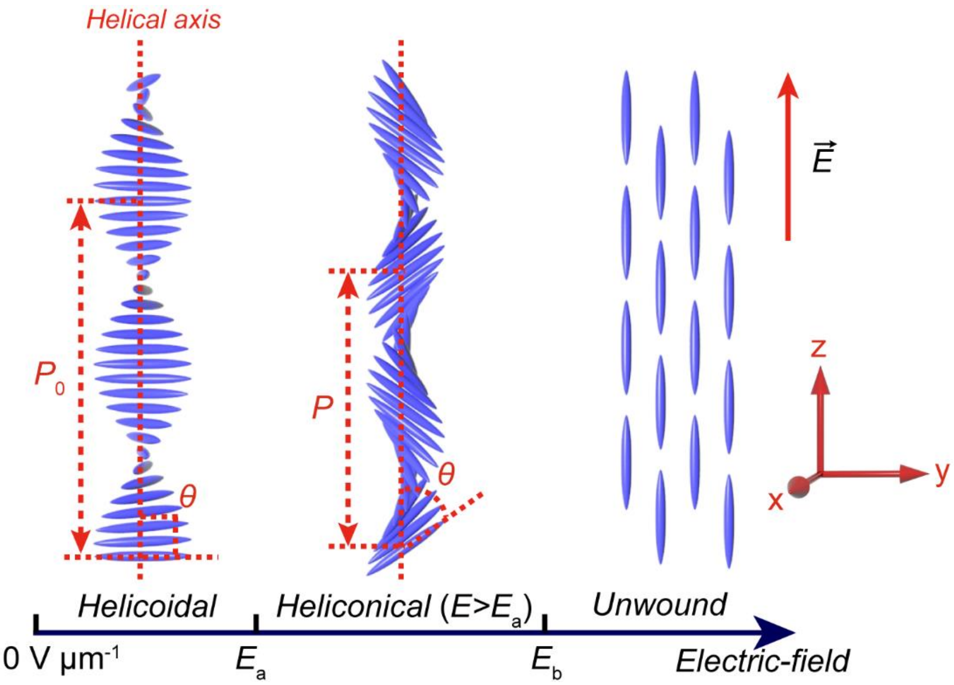

Supplementary Fig. 2 Schematic display of the director orientation in helicoidal, heliconical and unwound superstructures as a function of the electric field.

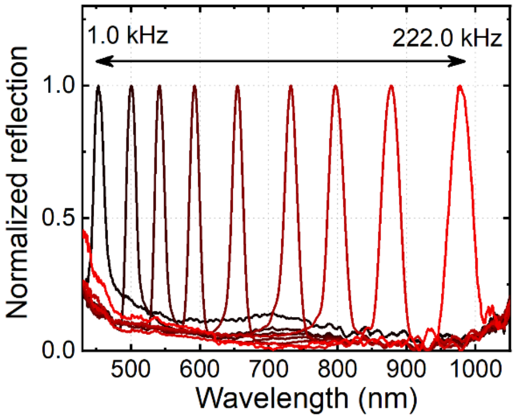

Supplementary Fig. 3 Wide dynamic spectral range of the reflection band obtained by modulating the frequency of the applied electric field from 1.0 kHz to 222.0 kHz at 0.52 V μm^{-1}. Source data are provided as a Source Data file.

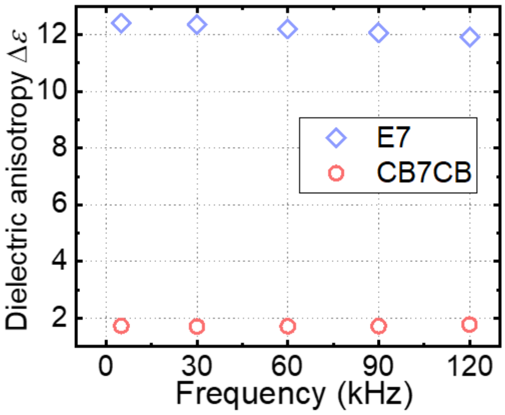

Supplementary Fig. 4 The nematic dielectric anisotropy Δε measurement results of pure CB7CB and E7 with increasing frequencies of electric fields, tested at 378.85 K (i.e., T_{TB}+1.6 K) and 298.15 K, respectively. The LC dimer CB7CB showed a uniaxial nematic (N) phase between 390.45 K (denoted as T_{NI}) and 377.25 K (T_{TB}), sandwiched between the isotropic and the twist-bend nematic phase (N_{tb}). Source data are provided as a Source Data file.

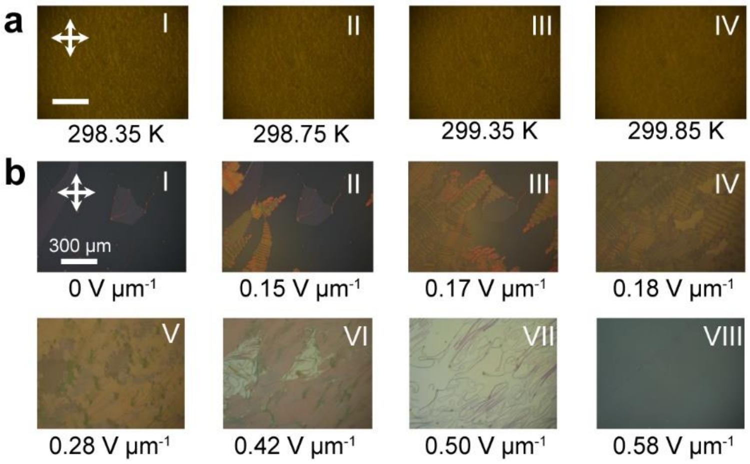

Supplementary Fig. 5 The POM textures under different stimuli. **a** The textures were almost invariable

with raising the temperature of the sample at a low electric field (0.08 V μm^{-1}) lower than the threshold. The actual temperature of the sample was obtained by IR thermography. **b** The textures of a common chiral LC under the increasing driving electric field at 1.0 kHz. The chiral LC consisted of 53.1 wt% nematic LC (E7), 43.4 wt% nematic LC (5CB), 3.5 wt% chiral agent (R811). The concentration ratio of E7 and 5CB was about 55 : 45， which was equal to that of E7 and CB7CB in our work. Increasing the electric field of the signal, the cholesteric phase gradually transited to the fingerprint texture decided by the comparison between the twisted elastic energy and electric field energy. Furthermore, the helical superstructure was unwound with the electric field arriving at 0.58 V μm^{-1}.

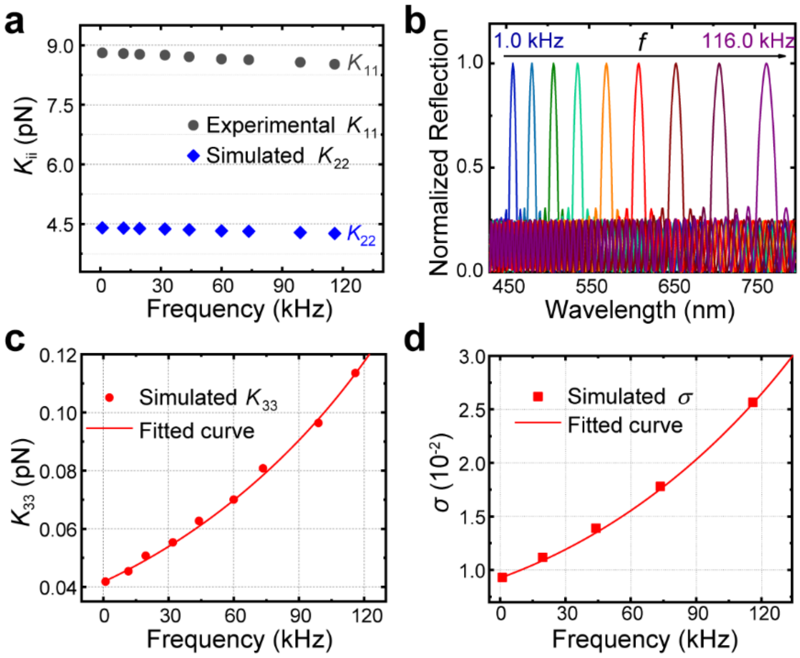

Supplementary Fig. 6 Frequency tuning performance of the elastic effects in the heliconical superstructure. **a** The experimentally measured splay elastic constant K_{11} at 0.52 V μm^{-1} of the heliconical superstructure as a function of the signal frequency and the twist elastic constant K_{22} was fitted as half of K_{11} with increasing frequency. **b** Simulated normalized selective reflection spectra in the heliconical superstructure corresponding to the frequency increasing from 1.0 kHz to 116.0 kHz at 0.52 V μm^{-1} with the Berreman's 4 × 4 matrix method. The specific value of frequency was consistent with that in Fig. 2b. For this measurement, ambient temperature was controlled at 298.15K. **c** The simulated bend elastic constant K_{33} increased as a function of the frequency at 0.52 V μm^{-1}. The solid line was the curve-fitting result based on Equation (S24). **d** Simulated σ increased as a function of the frequency at 0.52 V μm^{-1}. The solid line represented the curve-fitting results according to Equation (S25). Source data are provided as a Source Data file.

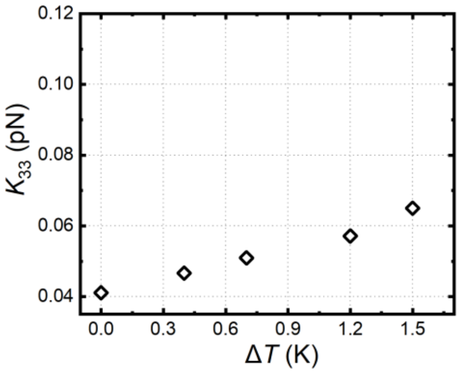

Supplementary Fig. 7 The simulated K_{33} of the heliconical system with the rising temperature at 1.0 kHz, 0.52 V μm^{-1}. The initial ambient temperature was 298.15K.

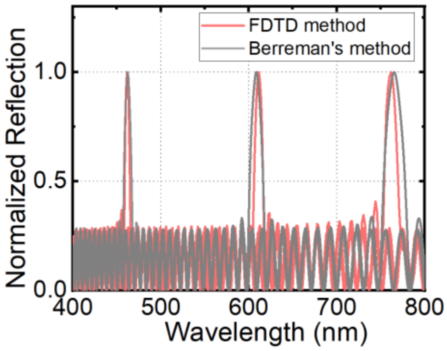

Supplementary Fig. 8 Simulated reflection spectra in the heliconical superstructure corresponding to different frequencies at 0.52 V μm^{-1} by the Berreman's 4×4 matrix method and FDTD method, respectively. Source data are provided as a Source Data file.

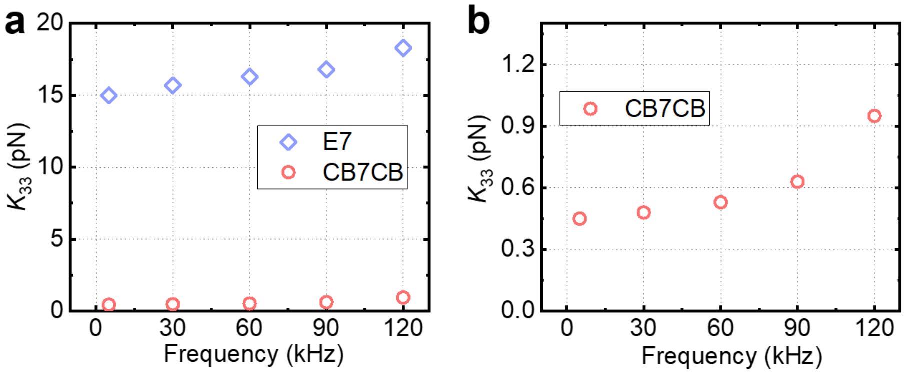

Supplementary Fig. 9 Bend elastic constants K_{33} measurement results of pure CB7CB and E7 in nematic phase under different frequencies of electric fields, tested at 378.85 K (i.e., T_{TB}+1.6 K) and 298.15 K, respectively. **a** The experimentally measured bend elastic constants of pure CB7CB and E7. **b** Expanded vertical scale for K_{33} of CB7CB. Source data are provided as a Source Data file.

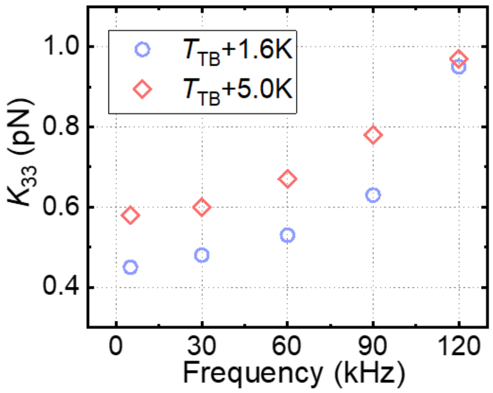

Supplementary Fig. 10 Bend elastic constants K_{33} of CB7CB in nematic phase with different frequencies of electric fields, measured at 378.85 K (i.e., T_{TB}+1.6 K) and 382.25 K (i.e., T_{TB}+5.0 K), respectively. Source data are provided as a Source Data file.

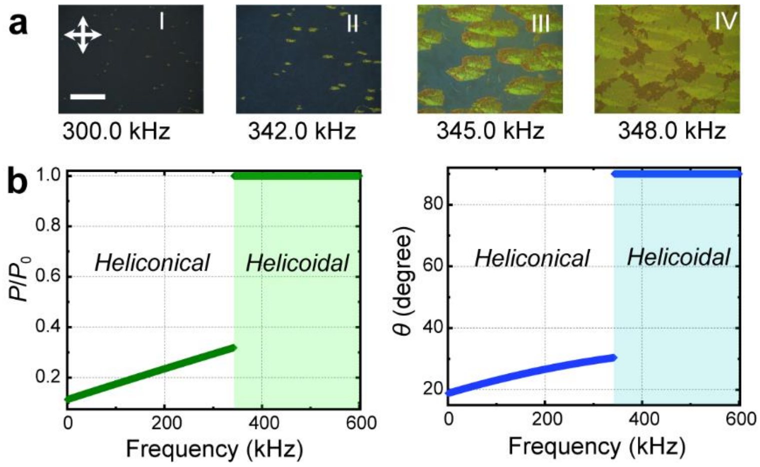

Supplementary Fig. 11 Frequency tuning performance of the heliconical superstructure. **a** The POM textures with increasing the frequency at 0.52 V μm^{-1}, indicating the heliconical superstructure transformed into helicoidal superstructure once the applied frequency exceeded 342.0 kHz. **b** Dependence of the simulated relative pitch length and the simulated oblique angle on the signal frequency from 1.0 kHz to 600.0 kHz at 0.52 V μm^{-1}. The oblique angle between 0° and 90° (with the relative pitch length between 0 and 1) corresponded to the heliconical superstructure, while the oblique angle of about 90° (with the relative pitch length of 1) corresponded to the helicoidal superstructure. As the frequency exceeded the stop-frequency (f_s= 342.0 kHz), the oblique angle jumped to 90° while the relative pitch length jumped to 1.

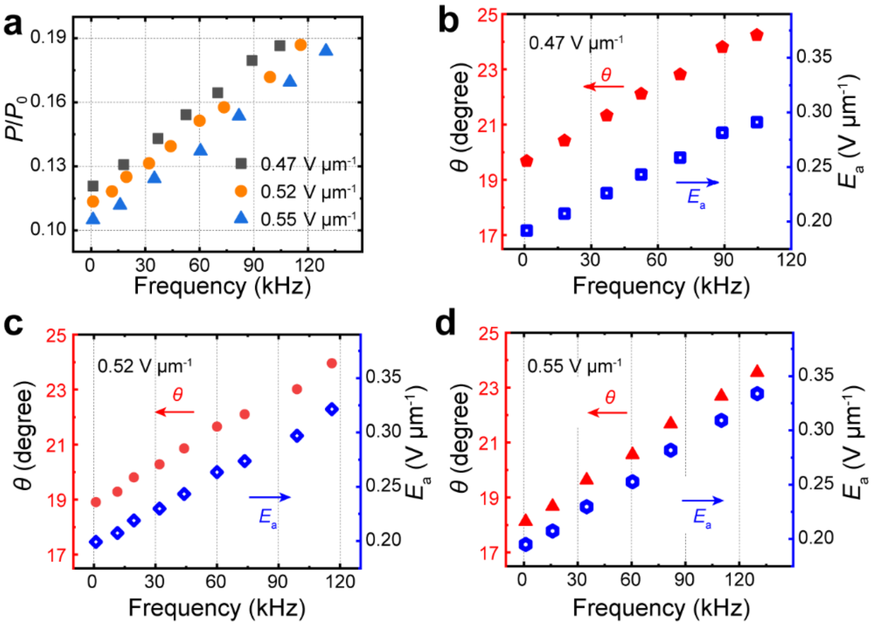

Supplementary Fig. 12 Performance of the heliconical superstructure with frequency tuning. **a** Dependence of the relative pitch length at 0.47 V μm^{-1}, 0.52 V μm^{-1} and 0.55 V μm^{-1}, respectively. Dependence of the oblique angle and threshold field E_a on the frequency at **b** 0.47 V μm^{-1}, **c** 0.52 V μm^{-1} and **d** 0.55 V μm^{-1}. Source data are provided as a Source Data file.

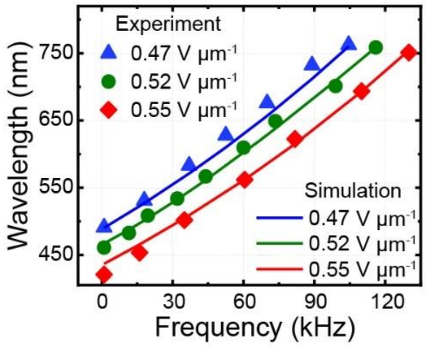

Supplementary Fig. 13 Frequency-dependent central wavelength of the experimental and simulated results based on Equation (S36).

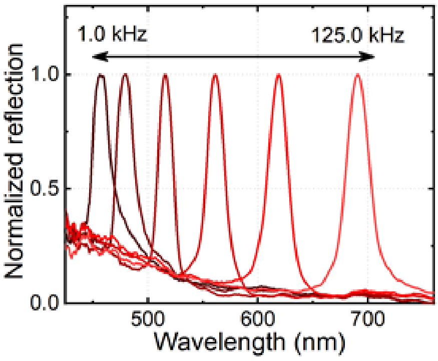

Supplementary Fig. 14 The spectral range of the reflection band obtained by modulating the

frequency of the applied electric field in the mixture containing nematic 5CB (the weight ratio was CB7CB : 5CB : R811 = 43.4 : 53.1 : 3.5). The electric field strength was 0.75 V μm^{-1}, and the temperature was 298.15 K.

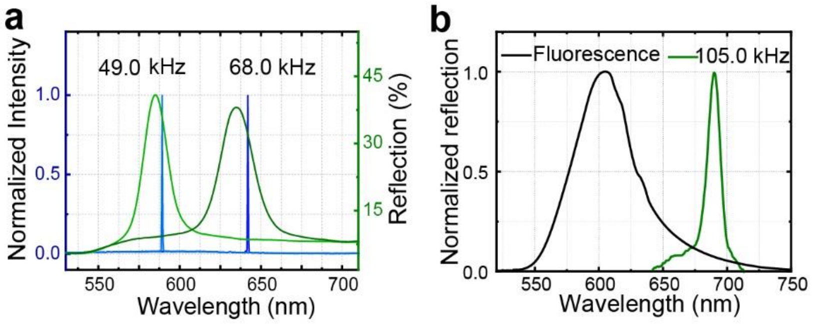

Supplementary Fig. 15 laser performance in heliconical LC through the frequency of electric signal. **a** The corresponding spectra of reflection band and laser emission with the 0.52 V μm^{-1} electric field. **b** The range of the reflection band in the heliconical structure went out of the fluorescent peak of laser dye due to the increase of the frequency to 105.0 kHz at 0.55 V μm^{-1}, leading to the disappearance of the laser emission.

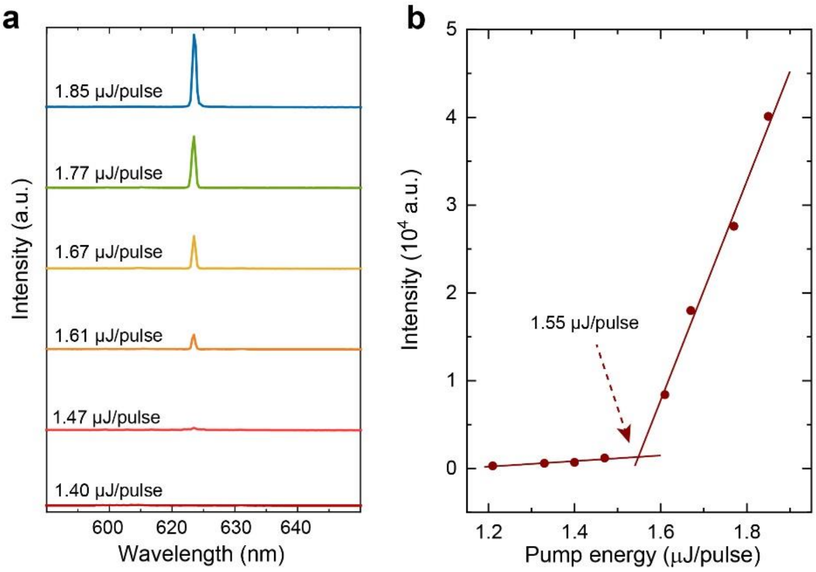

Supplementary Fig. 16 a The laser emission spectra and laser emission with the 0.52 V μm^{-1} electric field at 63.0 kHz. **b** Plots of the photoluminescence peak intensity vs pump energy and the laser thresholds was about 1.55 μJ/pulse.

II. Supplementary Methods

Optimal experiment

In order to achieve a wider dynamic spectral range of reflection band by modulating the signal frequency

at a lower electric field stimulation in the room temperature, the concentration of chiral agent R811 in the cholesteric liquid crystal (CLC) mixture was optimized. The mixture presented widest dynamic spectral range of reflection band when the concentration of R811 was maintained at 3.5 wt% giving an initial pitch P_0 about 2.6 μm with the frequency varying from 1.0 kHz to 116.0 kHz. Furthermore, the optimal concentration ratio of nematic liquid crystal (LC) E7 and the LC dimer CB7CB in the mixture was proved to be 55: 45 under a low electric field stimulation at the room temperature of 298.15 K experimentally (Supplementary Fig. 1).

Electrical experiment

Frequency-dependent dielectric permittivities were calculated from the capacitance-voltage measurements[1] on a planar cell (cell gap was 12.0 μm). The active area of the patterned ITO electrode was 165 mm². The perpendicular component of dielectric permittivity $\varepsilon_{\perp}$ was calculated from capacitance measurements at low voltages, below the Fréedericksz threshold, whereas the parallel component $\varepsilon_{/\!/}$ was determined by extrapolation method at high voltages. The dielectric anisotropy $\Delta\varepsilon$ of the material was defined as $\Delta\varepsilon=(\varepsilon_{/\!/}-\varepsilon_{\perp})$. The cell capacitance was measured by an LCR meter (HIOKI 3532-50). The dielectric anisotropy of pure CB7CB or E7 was almost independent on the frequency of electric field (Supplementary Fig. 4).

Theoretical framework about elastic constants

The phenomenon of reflection band shift responding to frequency was observed with a constant applied electric field in the experiment. Furthermore, the mechanism of such phenomenon was explored. The electrically induced heliconical superstructure[2] (also named oblique helicoidal structure) can be generated from a CLC system possessing a very small bend elastic constant K_{33}, due to the chiral character competing with dielectric torque, where the LC director (denoted as $\hat{n}$) arranged around the helical axis and formed an oblique angle with this axis. And the pitch length and oblique angle could be controlled by applying electric field parallel to the helical axis while the direction of the axis remained the same. However, the common liquid crystals (LCs) could not be utilized for the investigation into the transformation of the heliconical superstructure. Recently, it was suggested that the novel dimer LCs, with two rigid rod-like units connected by a flexible chain with an odd number of links such as CB7CB, had a sufficiently smaller bend elastic value of K_{33} than the twist value of K_{22}, which indicated a possibility in inducing the heliconical superstructure. The theoretical framework of the heliconical structure was established with the Frank-Oseen free energy function.

We could write the energy density for CLC as

$$f=\frac{1}{2}\left[K_{11}\left(\nabla\cdot\hat{n}\right)^2+K_{22}\left(\hat{n}\cdot\nabla\times\hat{n}-q_0\right)^2+K_{33}\left(\hat{n}\times\nabla\times\hat{n}\right)^2-\Delta\varepsilon\varepsilon_0\left(\hat{n}\cdot\vec{E}\right)^2\right] \quad \text{(Equation S1)}$$

where K_{11}, K_{22} and K_{33} were the splay, twist, and bend elastic constant, respectively; $q_0 = 2\pi / P_0$, standing for the chirality, P_0 was the initial pitch; $\Delta\varepsilon=\varepsilon_{\parallel}-\varepsilon_{\perp}>0$ was the dielectric anisotropy, denoting the difference between the dielectric constant parallel and perpendicular to the director $\hat{n}$; ε_0 was the permittivity of vacuum; $\vec{E}$ was the applied electric field. Supposed that the helical axis and the electric field were both parallel to the z axis, $\vec{E}=\left(0,0,E\right)$. Minimize the free energy and we could deduce that

the LC molecules self-organize into the right-angle, helicoidal superstructure (i.e., common helical structure) in the absent of applied electric field (Supplementary Fig. 2). The director $\hat{n}$ could be described as:

$$n = \left(\cos\left(\frac{2\pi}{P_0} z \right), \sin\left(\frac{2\pi}{P_0} z \right), 0 \right) \quad \text{(Equation S2)}$$

Above some threshold field E_a, the helicoidal structure transformed into the heliconical state where the director twists around the helical axis with an oblique angle θ ($0 < \theta < \pi / 2$). The director $\hat{n}$ could be written as:

$$n = \left(\cos\left(\frac{2\pi}{P} z \right) \sin\theta, \sin\left(\frac{2\pi}{P} z \right) \sin\theta, \cos\theta \right) \quad \text{(Equation S3)}$$

When the electric field were much higher than the unwinding threshold field E_b, the director was parallel to the electric field due to its positive dielectric anisotropy. Here, the director $\hat{n}$ was given by:

$$n = (0,0,1) \quad \text{(Equation S4)}$$

The threshold field E_b could be calculated with:[2]

$$E_b = \frac{2\pi}{P_0} \frac{K_{22}}{\sqrt{\Delta\varepsilon\varepsilon_0 K_{33}}} \quad \text{(Equation S5)}$$

Decreasing the electric field below E_b, the unwound state transformed into a heliconical state with the director following Equation (S2). And the heliconical pitch length P was described as:[2]

$$P = \frac{2\pi}{E} \sqrt{\frac{K_{33}}{\varepsilon_0 \Delta\varepsilon}} \quad \text{(Equation S6)}$$

Minimizing the free energy function based on the density (Equation S1), the oblique angle θ can be calculated by:

$$\sin^2\theta = \frac{\sigma}{1-\sigma} \left(\frac{E_b}{E} - 1 \right) \quad \text{(Equation S7)}$$

where $\sigma = K_{33} / K_{22}$. The threshold field E_a could be deduced by balancing the energies of the right-angle and heliconical state in the external field, in the case of a small σ:

$$E_a \approx E_b \frac{\sigma\left[2+\sqrt{2(1-\sigma)}\right]}{1+\sigma} \quad \text{(Equation S8)}$$

Analysis and calculations

the frequency responsive spectrum of the heliconical structure in Fig.2 was measured from 460 nm to 760 nm mainly covering the visible reflection band for conveniently intuitive observation and practical application. In fact, its reflection band could shift from 460 nm to nearly 1000 nm by further increasing the frequency to about 222.0 kHz (Supplementary Fig. 3). The specific frequency-dependency of the heliconical structure with a continuous dynamic modulation on reflection color naturally inspired us to consider possible factors. The heliconical structure and the dynamic photonic band gap (PBG) shifting would be induced by appropriately alternating electric field, which was mainly attributed to the competition between twist and the dielectric torque in the chiral LC system with quite small K_{33}. The sufficiently small K_{33} was obtained owing to the bridging alkyl chain between two rod-like cyanobiphenyl moieties in CB7CB[3,4] in the mixture. Instead of forming the heliconical structure with spectral modulation of the selective reflection band under electric field modulation, the helicoidal structure of the mixture gradually transited to the fingerprint texture and finally unwound as the electric field increased (Supplementary Fig. 5b), when CB7CB was replaced with a room temperature nematic LC 4-n-Pentyl-4-cyanobiphenyl (5CB) possessing a high bend elastic constant K_{33}. The parameters of 5CB were listed below: dielectric anisotropy $\Delta\varepsilon$ =13.30, bend elastic constant K_{33} = 8.25 pN at 298.15 K.

Based on Equation (S33), the band shift could be achieved by the possible change of bend elastic constant in the mixture. Therefore, considering the temperature dependent of elastic constants in CB7CB[5] and its mixture[6], and the dielectric heating effects of LCs in an ac electric field[7], we measured the temperature change during the frequency driving of the mixture. An imperceptible rising of sample temperature for only about 1.50 K was detected after applying a 116.0 kHz signal at 0.52 V μm^{-1} across LC cell. Nevertheless, the slight heating was actually not the only factor inducing reflection band shift. Supplementary Fig. 5a indicated an almost invariable optical texture when changing the sample temperature by a hot stage for 1.50 K, while applying an electric field lower than the threshold, E_a, implying a delicate inherent coupling among the strength of electric field, the frequency of applied signal, and the elastic effect of LC system. Furthermore, enhancing the electric field strength to 0.52 V μm^{-1} at 1.0 kHz and controlling the temperature to raise by 1.50 K, the spectrum showed a certain shift but the range of band shift was less than half of it in Fig. 2c. Therefore, high-frequency thermal effect was actually not the main reason for the broad dynamic spectral range.

To elucidate such coupling, the frequency- and temperature-dependent elastic effects and the reflection spectra were deduced combining experimental results and the classical elastic energy model. Prior work indicated a significant dependency of decreasing trend of K_{11} with temperature of CB7CB and wherein the ratio K_{11} / K_{22} was more or less constant.[5] Besides, Dozov reported that the twist-bend phase was stable when $K_{11} > 2K_{22}$.[8] Consequently, we assumed that K_{22} was always half of K_{11} with increasing frequency in the heliconical structure. Experimentally, the dependence of the splay elastic constant K_{11} on the signal frequency at 0.52 V μm^{-1} was obtained. Then, the assumed K_{22} was obtained as half of experimental K_{11} (Supplementary Fig. 6a).

Based on Equations (S1)-(S8) and the Berreman's 4 × 4 matrix method[9,10](detailed description was shown in Equations S9-S20), the reflection spectra with the signal frequency increasing at 0.52 V μm^{-1} (Supplementary Fig. 6b) were simulated, which were accompanied by calculating the appropriate K_{33}. According to calculating the reflection band based on the experimental results, K_{33} with increasing the frequency was obtained (Supplementary Fig. 6c). The simulated reflection spectra were almost consistent with the experimental spectra shown in Fig. 2. Furthermore, the ratio $\sigma = K_{33} / K_{22}$ as frequency changed (Supplementary Fig. 6d) were obtained. According to Supplementary Fig. 5c, the bend elastic constant of the mixture changed greatly (i.e., around 170%) with the increase of frequency to achieve broadband reflection band modulation. While the bend elastic constant (Supplementary Fig. 7) changed less than half through directly raising the same temperature as the thermal effect in Fig. 2c with maintaining the frequency at 1.0 kHz. Consequently, the bend elastic effect of the mixture was not only affected indirectly by high-frequency heating, but also presented a direct influence from the frequency.

Additionally, The finite-difference time-domain (FDTD) method[11] was also applied to simulate the reflection spectra of the heliconical system and showed a satisfactory agreement with the results obtained by Berreman 4×4 matrix method, indicating the feasibility of the Berreman 4×4 matrix method for optical analysis in the heliconical structure (Supplementary Fig. 8).

To further verify the results, the bend elastic constants K_{33} of pure CB7CB and E7 in nematic phase were determined at different frequencies according to the Capacitance-Voltage method (detailed in Equations S21-S23), which was an effective method to measure LC parameters and was widely used to measure the bend elastic constant K_{33} of nematic LCs and LC dimer materials, such as CB7CB[5,6,12]. The LC dimer CB7CB showed a uniaxial nematic (N) phase between 390.45 K (denoted as T_{NI}) and 377.25 K (T_{TB}), sandwiched between the isotropic and the twist-bend nematic phase (N_{tb}). The CB7CB were measured at the temperature T= (T_{TB}+1.6) K, while E7 was measured at 298.15 K in the nematic phase. K_{33} of CB7CB was 0.45 pN at 5.0 kHz and was 0.95 pN at 120.0 kHz, where the bend elastic constant approximately increased by around 111% with increasing frequency (Supplementary Fig. 9). Our tested result of initial K_{33} at 5.0 kHz with the Capacitance-Voltage method was consistent with the results measured by dynamic light scattering method at the same temperature[1], which demonstrated the validity of the method for measuring K_{33}. Meanwhile, K_{33} of E7 was a large constant 15.0 pN at 5.0 kHz and the bend elastic constant approximately increased by 22% in the same frequency range (Supplementary Fig. 9a). Consequently, played a dominant role to the reduction of K_{33} in the mixture, and ensured the formation and stability of such a heliconical system during the modulation of frequency, and therefore presenting a frequency controllable band shift of the reflection within a broad spectral range.

Furthermore, considering that K_{33} of CB7CB exhibited temperature dependence, and the temperature (T_{TB}+1.6 K) for measuring K_{33} of CB7CB from 5.0 kHz to 120.0 kHz was close to phase transition temperature (T_{TB}), K_{33} of CB7CB with the increase of frequency was measured at higher temperature

(i.e., T_{TB}+5.0 K) (Supplementary Fig. 10). The change value of K_{33} (i.e., around 64%) with increasing frequency at T_{TB}+5.0 K was smaller than that (i.e., around 111%) at $T= T_{TB}$+1.6 K. Namely, K_{33} of CB7CB exhibited more significant rising with the increase of the frequency as the temperature was closer to the phase transition between N_{tb} and N phase.

Calculation parameters were listed below: initial pitch P_0 = 2.60 μm, birefringence n_e=1.721, n_o=1.555, dielectric anisotropy $\varepsilon_{\parallel}$=13.06, $\varepsilon_{\perp}$=5.14, $\Delta\varepsilon$ =7.92, which had been measured in our material system.

The detailed calculation process of the Berreman's 4 × 4 matrix method was shown below. Considering an optical film of a uniaxial CLC, which was in heliconical superstructure, the CLC director was

$$\vec{n}=\begin{pmatrix} n_x \\ n_y \\ n_z \end{pmatrix}^T=\begin{pmatrix} \cos\left(\frac{2\pi}{P}\right)\sin\theta \\ \sin\left(\frac{2\pi}{P}\right)\sin\theta \\ \cos\theta \end{pmatrix}^T \qquad \text{(Equation S9)}$$

For normal incident light, the dielectric tensor was

$$\vec{\vec{\varepsilon}}=\begin{pmatrix} \varepsilon_{\perp}+\Delta\varepsilon n_x^2 & \Delta\varepsilon n_x n_y & \Delta\varepsilon n_x n_z \\ \Delta\varepsilon n_x n_y & \varepsilon_{\perp}+\Delta\varepsilon n_y^2 & \Delta\varepsilon n_y n_z \\ \Delta\varepsilon n_x n_z & \Delta\varepsilon n_y n_z & \varepsilon_{\perp}+\Delta\varepsilon n_z^2 \end{pmatrix} \qquad \text{(Equation S10)}$$

Where $\varepsilon_{\perp}=n_o^2$ and $\Delta\varepsilon=\varepsilon_{\parallel}-\varepsilon_{\perp}=n_e^2-n_o^2$. The Berreman matrix was

$$\vec{\vec{Q}}=\frac{1}{\left(\varepsilon_{\perp}+\Delta\varepsilon n_z^2\right)}\cdot \begin{pmatrix} 0 & \varepsilon_{\perp}+\Delta\varepsilon n_z^2 & 0 & 0 \\ \varepsilon_{\perp}\left[\varepsilon_{\perp}+\Delta\varepsilon\left(n_x^2+n_z^2\right)\right] & 0 & \varepsilon_{\perp}\Delta\varepsilon n_x n_y & 0 \\ 0 & 0 & 0 & \varepsilon_{\perp}+\Delta\varepsilon n_z^2 \\ \varepsilon_{\perp}\Delta\varepsilon n_x n_y & 0 & \varepsilon_{\perp}\left(\varepsilon_{\perp}+\Delta\varepsilon n_z^2\right)+\varepsilon_{\perp}\Delta\varepsilon n_y^2 & 0 \end{pmatrix} \qquad \text{(Equation S11)}$$

The refractive index n of the isotropic medium outside the CLC film was assumed to be 1. On top of the cholesteric film, there is incident light and reflected light, and the actual Berreman vector was the sum of the Berreman vectors of the incident light and reflected light. For the incident light, the Berreman vector was

$$\vec{\psi}_i^T = \begin{pmatrix} E_{xi} & E_{xi} & E_{yi} & E_{yi} \end{pmatrix} \quad \text{(Equation S12)}$$

Where E was the electric field components of the transmitted and reflected light. For the reflected light with an opposite direction of propagation, the Berreman vector was

$$\vec{\psi}_r^T = \begin{pmatrix} E_{xr} & -E_{xr} & E_{yr} & -E_{yr} \end{pmatrix} \quad \text{(Equation S13)}$$

At the bottom of the cholesteric film, there was only the transmitted light whose Berreman vector was

$$\vec{\psi}_t^T = \begin{pmatrix} E_{xt} & E_{xt} & E_{yt} & E_{yt} \end{pmatrix} \quad \text{(Equation S14)}$$

We divided the CLC film into N slabs with thicknesses Δz. The Berreman vectors at the boundaries between the slabs were:

$$\vec{\psi}(0) = \vec{\psi}_i + \vec{\psi}_r \quad \text{(Equation S15)}$$

$$\vec{\psi}(1) = \vec{P}(z_1) \cdot \vec{\psi}(0) \quad \text{(Equation S16)}$$

$$\vec{\psi}(2) = \vec{P}(z_2) \cdot \vec{\psi}(1) = \vec{P}(z_2) \cdot \vec{P}(z_1) \cdot \vec{\psi}(0) \quad \text{(Equation S17)}$$

$$\vdots$$

$$\vec{\psi}_t = \vec{\psi}(N) = \prod_{i=1}^{N} \vec{P}(z_i) \cdot \vec{\psi}(0) = \prod_{i=1}^{N} \vec{P}(z_i) \cdot (\vec{\psi}_i + \vec{\psi}_r) \quad \text{(Equation S18)}$$

where the states $\vec{P}$ for each slab could be numerically calculated by fast Berreman method.[13] Solving the above Equations, the outgoing transmitted light $\vec{\psi}_t$ and outgoing reflected light $\vec{\psi}_r$ can be obtained. Furthermore, the reflectance could be acquired by:

$$R = \frac{E_{xr}^2 + E_{yr}^2}{E_{xi}^2 + E_{yi}^2} \quad \text{(Equation S19)}$$

Therefore the transmittance could be calculated by:

$$T = \frac{E_{xt}^2 + E_{yt}^2}{E_{xi}^2 + E_{yi}^2} \quad \text{(Equation S20)}$$

The cell capacitance C was plotted as a function of applied voltage V to find the splay Fréedericksz threshold voltage (V_{th}) by the double-line extrapolation method. The Fréedericksz threshold V_{th} was given explicitly by

$$V_{\mathrm{th}} = \pi\sqrt{\frac{K_{11}}{\varepsilon_0 \Delta\varepsilon}} \quad \text{(Equation S21)}$$

where ε_0 is the vacuum permittivity, $\Delta\varepsilon=(\varepsilon_{/\!/}-\varepsilon_{\perp})$ is the dielectric anisotropy of the material, and K_{11} is the splay elastic constant. In order to calculate bend elastic constant K_{33}, the voltage-capacitance curve was fitted to the following expression[14]:

$$\frac{C}{C_0} = \frac{2}{\pi}\sqrt{1+\gamma\sin^2\theta_m}\,\frac{V_{\mathrm{th}}}{V}\int_{\theta_0}^{\theta_m}\sqrt{\frac{\left(1+\gamma\sin^2\theta\right)\left(1+\kappa\sin^2\theta\right)}{\sin^2\theta_m-\sin^2\theta}}\,d\theta \quad \text{(Equation S22)}$$

$$\frac{V}{V_{\mathrm{th}}} = \frac{2}{\pi}\sqrt{1+\gamma\sin^2\theta_m}\int_{\theta_0}^{\theta_m}\sqrt{\frac{1+\kappa\sin^2\theta}{\left(1+\gamma\sin^2\theta\right)\left(\sin^2\theta_m-\sin^2\theta\right)}}\,d\theta \quad \text{(Equation S23)}$$

Where C_o is the zero-field capacitance, θ is the angle between the director and the substrate , θ_m is the maximum angle in the middle of the cell, θ_0 is the pretilt angle, $\gamma=\varepsilon_{/\!/}/\varepsilon_{\perp}-1$, $\kappa = K_{33}/K_{11}-1$. Capacitance measurements were carried out at different frequencies of electric fields using an LCR meter (HIOKI 3532-50). Furthermore, K_{33} can be determined from a numerical fit of capacitance-voltage to Equations (S21)-(S23).

As shown in Supplementary Figure 5c, exponential fitting was employed to the frequency-dependent K_{33} when the electric field was 0.52 V μm^{-1}:

$$K_{33}=A\exp\left(B\frac{f}{f_s}\right) \quad \text{(Equation S24)}$$

Where the fitting parameter A = 0.033 pN, representing the theoretical value of K_{33} when the alternating electric field was switched to direct electric field. B = 2.950 was a fitting parameter. Here, $f < f_s$, f was the applied signal frequency. f_s = 342.0 kHz was defined as stop-frequency, meaning that the heliconical structure would transform into the helicoidal structure once the frequency exceeded the stop-frequency f_s (Supplementary Fig. 11).

Then the frequency-dependent σ was obtained and the similar exponential equation was employed to fit the relationship when the electric field was 0.52 V μm^{-1} (Supplementary Fig. 6d):

$$\sigma(f) = C\exp\left(D\frac{f}{f_s}\right)$$ (Equation S25)

where the fitting parameter D = 3.000, while C = 0.009 represents the theoretical value of σ when the alternating electric field was switched to direct electric field.

The transformation between different superstructures were accompanied by pitch length and oblique angle as the frequency increased at a constant electric field ($E_a<E<E_b$). The CLC system exhibits the heliconical structure with the corresponding relative pitch length $P/P_0 < 1$ and oblique angle θ between 0° and 90° at the initial frequency of 1.0 kHz. The relative pitch length gradually increased to about 0.32 and oblique angle increased to around 30° when the frequency increased approaching the stop-frequency f_s. As long as the frequency exceeded the stop-frequency f_s, the CLC system transformed into the helicoidal structure. Meanwhile, the oblique angle jumped to 90° while the pitch length shown the same trend (Supplementary Fig. 11). Experimentally, the heliconical structure showing a uniform state was observed when the signal frequency varied from 1.0 kHz to 342.0 kHz (i.e., the experimental stop-frequency, which approximated to the fitted result) at 0.52 V μm^{-1}. When the frequency exceeded 342.0 kHz, the texture gradually transformed into a focal conic texture, indicating the helicoidal structure.

Combining the Equations (S5)-(S8) with (S24) and (S25), the threshold field E_b, E_a, the pitch length P and the oblique angle θ can be described with the dependence of frequency:

$$E_b(f) = \frac{2\pi}{P_0\sqrt{\Delta\varepsilon\varepsilon_0}}\frac{\sqrt{K_{33}(f)}}{\sigma(f)}$$ (Equation S26)

$$E_a(f) \approx E_b(f)\frac{\sigma(f)\left[2+\sqrt{2(1-\sigma(f))}\right]}{1+\sigma(f)}$$ (Equation S27)

$$P(f) = \frac{2\pi}{E}\sqrt{\frac{K_{33}(f)}{\varepsilon_0\Delta\varepsilon}}$$ (Equation S28)

$$\sin^2\theta(f) = \frac{\sigma(f)}{1-\sigma(f)}\left(\frac{E_b(f)}{E}-1\right)$$ (Equation S29)

Furthermore, the relative pitch length, the oblique angle and threshold field E_a at different electric fields were also acquired as the frequency increased at 298.35 K (Supplementary Fig. 12), which were all positively related to frequency.

Besides reflection spectra simulation with the Berreman's 4 × 4 matrix method, the central wavelength of reflection band from the heliconical structure can be approximately described as:

$$\lambda_c \approx \bar{n}P$$ (Equation S30)

In which,

$$\bar{n} = \sqrt{\frac{2n_o^2 + n_{e\cdot eff}^2}{3}} \qquad \text{(Equation S31)}$$

$$n_{e\cdot eff} = \frac{n_e n_o}{\sqrt{n_e^2 \cos^2\theta + n_o^2 \sin^2\theta}} \qquad \text{(Equation S32)}$$

$\bar{n}$ is the average refractive index and $n_{e\cdot eff}$ is the effective extraordinary index. Considering the Equations above, the reflection central wavelength in Equation (S30) can be denoted as:

$$\lambda_c \approx \frac{2\pi\bar{n}\sqrt{K_{33}}}{E\sqrt{\varepsilon_0 \Delta\varepsilon}} \qquad \text{(Equation S33)}$$

Combined with Equations (S24) and (S29) about the frequency-dependent $K_{33}(f)$ and $\sin^2\theta(f)$, $n_{e\cdot eff}$, $\bar{n}$ and the central wavelength λ_c in Equations (S31)-(S33) can be expressed as:

$$n_{e\cdot eff}(f) = \frac{n_e n_o}{\sqrt{n_e^2 + \left(n_o^2 - n_e^2\right)\sin^2\theta(f)}} \qquad \text{(Equation S34)}$$

$$\bar{n}(f) = \sqrt{\frac{2n_o^2 + \left[n_{e\cdot eff}(f)\right]^2}{3}} \qquad \text{(Equation S35)}$$

$$\lambda_c(f) \approx \frac{2\pi\bar{n}(f)\sqrt{K_{33}(f)}}{E\sqrt{\varepsilon_0 \Delta\varepsilon}} \qquad \text{(Equation S36)}$$

In order to verify the equation of frequency-dependent λ_c, the simulated central wavelengths were calculated at different electric fields (i.e., 0.47 V μm^{-1}, 0.52 V μm^{-1} and 0.55 V μm^{-1}), which were agreed well with the experimental results (Supplementary Fig. 13).

The similar frequency dependency of the reflection spectrum was observed as the nematic LC used herein was replaced by another nematic LC 5CB with the positive dielectric constant (Supplementary Fig. 14). Therefore, we predicted that such frequency-dependency may be an intrinsic characteristic of the heliconical arranged system, rather caused by the common dielectric anisotropy of the system. The weight ratio of the mixture was CB7CB:5CB:R811=43.4:53.1:3.5. It could form the heliconical structure with the electric field of 1.0 kHz and 0.75 V μm^{-1} at 298.15 K.

Laser performance characterization

The laser was emitted at the long wavelength band-edge, when the reflection band from the heliconical structure was in the fluorescent spectrum range of the laser dye (DCM). The laser emission in heliconical system had been demonstrated in previous paper[15]. Here, the laser emission was right-circularly polarized, consistent with the chirality of the heliconical structure. The laser emission would disappear when the reflection band went out of the fluorescent range as the frequency increased to 105.0 kHz at 0.55 V μm^{-1} (Supplementary Fig. 15). Testing with 3 other samples obtained the same results. The laser emission still occurred after ten hours.

To obtain the threshold of the laser emission, the intensity was measured when the wavelength of laser emission was about 623 nm (Supplementary Fig. 16). The emission intensity enhanced with the pumping energy increased. Therefore, the laser thresholds could be determined as 1.55 μJ/pulse.

References

1. Babakhanova, G. et al. Elastic and viscous properties of the nematic dimer CB7CB. *Phys. Rev. E* **96**, 062704 (2017).
2. Meyer, R. B. Effects of Electric and Magnetic Fields on the Structure of Cholesteric Liquid Crystals. *Appl. Phys. Lett.* **12**, 281-282 (1968).
3. Borshch, V. et al. Nematic twist-bend phase with nanoscale modulation of molecular orientation. *Nat. Commun.* **4**, 2635 (2013).
4. Cestari, M. et al. Phase behavior and properties of the liquid-crystal dimer 1",7"-bis(4-cyanobiphenyl-4'-yl) heptane: a twist-bend nematic liquid crystal. *Phys. Rev. E: Stat., Nonlinear, Soft Matter Phys.* **84**, 031704 (2011).
5. Yun, C.-J., Vengatesan, M. R., Vij, J. K. & Song, J.-K. Hierarchical elasticity of bimesogenic liquid crystals with twist-bend nematic phase. *Appl. Phys. Lett.* **106**, 173102 (2015).
6. Sridurai, V., Kanakala, M. B., Yelamaggad, C. V. & Nair, G. G. Effect of gelation on the Frank elastic constants in a liquid crystalline mixture exhibiting a twist bend nematic phase. *Soft Matter* **15**, 9982-9990 (2019).
7. Yin, Y., Shiyanovskii, S. V. & Lavrentovich, O. D. Electric heating effects in nematic liquid crystals. *J. Appl. Phys.* **100**, 024906 (2006).
8. Dozov, I. On the spontaneous symmetry breaking in the mesophases of achiral banana-shaped molecules. *Europhys. Lett.* **56** (2001).
9. Yuan, C.-l. et al. Stimulated transformation of soft helix among helicoidal, heliconical, and their inverse helices. *Sci. Adv.* **5**, eaax9501 (2019).
10. Berreman, D. W. Optics in Stratified and Anisotropic Media: 4×4-Matrix Formulation. *J. Opt. Soc. Am.* **62**, 502-510 (1972).
11. Bregar, A., Štimulak, M. & Ravnik, M. Photonic properties of heliconical liquid crystals. *Opt. Express* **26**, 23265-23277 (2018).
12. Basu, R. Enhancement of polar anchoring strength in a graphene-nematic suspension and its effect on nematic electro-optic switching. *Phys. Rev. E* **96**, 012707 (2017).
13. Wöhler, H., Haas, G., Fritsch, M. & Mlynski, D. Faster 4× 4 matrix method for uniaxial inhomogeneous media. *J. Opt. Soc. Am.* ***A*** **5**, 4 (1988).
14. Walton, H. G. Influence of TiO2 Nanoparticle Doping on the Splay and Bend Elastic Constants of the Nematic Liquid Crystal 4′-butyl-4-heptyl-bicyclohexyl-4- carbononitrile, CCN47. *Mol. Cryst. Liq. Cryst.* **574**, 60-66 (2013).

15. Xiang, J. et al. Electrically tunable laser based on oblique heliconical cholesteric liquid crystal. *Proc. Natl. Acad. Sci. U.S.A.* **113**, 12925-12928 (2016).
