# Supplementary material for: Dynamically actuated soft heliconical architecture via frequency of electric fields
Source: Nat Commun. 2022 May 17;13:2712. doi: 10.1038/s41467-022-30486-2 (PMC9114134; doi:10.1038/s41467-022-30486-2)
Supplement: Supplementary file 2 — Description of Additional Supplementary Files [file 41467_2022_30486_MOESM2_ESM.pdf]

Description of Additional Supplementary Files

File Name: Supplementary Movie 1
Description: Dynamics of frequency-actuated heliconical soft architecture.

File Name: Supplementary Movie 2
Description: Dynamic responsiveness on both the electricfield strength and the frequency.

File Name: Supplementary Movie 3
Description: The decoding process of the information encoder.
