# Supplementary material for: Dynamically actuated soft heliconical architecture via frequency of electric fields
Source: Nat Commun. 2022 May 17;13:2712. doi: 10.1038/s41467-022-30486-2 (PMC9114134; doi:10.1038/s41467-022-30486-2)
Supplement: Supplementary file 6 — Lasing Reporting Summary [file 41467_2022_30486_MOESM6_ESM.pdf]

nature research

Corresponding author(s): Quan Li

Lasing Reporting Summary

Nature Research wishes to improve the reproducibility of the work that we publish. This form is intended for publication with all accepted papers reporting claims of lasing and provides structure for consistency and transparency in reporting. Some list items might not apply to an individual manuscript, but all fields must be completed for clarity.

For further information on Nature Research policies, including our data availability policy, see Authors & Referees.

Experimental design

Please check: are the following details reported in the manuscript?

1. Threshold

Plots of device output power versus pump power over a wide range of values indicating a clear threshold

☒ Yes ☐ No — Supplementary Fig. 16b

2. Linewidth narrowing

Plots of spectral power density for the emission at pump powers below, around, and above the lasing threshold, indicating a clear linewidth narrowing at threshold

☒ Yes ☐ No — Supplementary Fig. 16a

Resolution of the spectrometer used to make spectral measurements

☒ Yes ☐ No — Characterization section in Methods.

3. Coherent emission

Measurements of the coherence and/or polarization of the emission

☒ Yes ☐ No — Laser performance characterization section in Supplementary Information.

4. Beam spatial profile

Image and/or measurement of the spatial shape and profile of the emission, showing a well-defined beam above threshold

☒ Yes ☐ No — Figure 5b,c in the main text.

5. Operating conditions

Description of the laser and pumping conditions
Continuous-wave, pulsed, temperature of operation

☒ Yes ☐ No — Lasing emission section in Methods.

Threshold values provided as density values (e.g. W cm^{-2} or J cm^{-2}) taking into account the area of the device

☒ Yes ☐ No — Supplementary Fig. 16b

6. Alternative explanations

Reasoning as to why alternative explanations have been ruled out as responsible for the emission characteristics
e.g. amplified spontaneous, directional scattering; modification of fluorescence spectrum by the cavity

☐ Yes ☒ No — We have analyzed the results and cited the relevant papers to demonstrate laser emission in heliconical system in main text and Supplementary Information.

7. Theoretical analysis

Theoretical analysis that ensures that the experimental values measured are realistic and reasonable
e.g. laser threshold, linewidth, cavity gain-loss, efficiency

☒ Yes ☐ No — Results and Discussion section.

8. Statistics

Number of devices fabricated and tested

☒ Yes ☐ No — Laser performance characterization section in Supplementary Information.

Statistical analysis of the device performance and lifetime (time to failure)

☒ Yes ☐ No — Laser performance characterization section in Supplementary Information.
